# OX40 signaling is involved in the autoactivation of CD4^+^CD28^−^ T cells and contributes to the pathogenesis of autoimmune arthritis

**DOI:** 10.1186/s13075-017-1261-9

**Published:** 2017-03-21

**Authors:** Juean Jiang, Cuiping Liu, Mi Liu, Yu Shen, Xiaohan Hu, Qin Wang, Jian Wu, Min Wu, Qi Fang, Xueguang Zhang

**Affiliations:** 1 Jiangsu Institute of Clinical Immunology & Jiangsu Key Laboratory of Clinical Immunology, First Affiliated Hospital of Soochow University, No. 708 Renmin Road, Suzhou, 215006 Jiangsu China; 2 Departments of Rheumatology, Third Affiliated Hospital of Soochow University, No. 185 Juqian Road, Changzhou, 213003 Jiangsu China

**Keywords:** Rheumatoid arthritis, OX40, CD4^+^CD28^−^ T cells

## Abstract

**Background:**

CD4^+^CD28^−^ T cells exhibit autoreactive potential in autoimmune disorders, including rheumatoid arthritis (RA). It is not well known which costimulator functions as an alternative second signal in the activation of this subset after CD28 expression is downregulated. Tumor necrosis factor receptor superfamily member OX40 is a key costimulator in the activation of T cells. The aim of this study was to investigate the costimulatory effects of OX40 on CD4^+^CD28^−^ T cells in autoimmune arthritis.

**Methods:**

Clinical samples were collected from patients with RA and control subjects. Collagen-induced arthritis (CIA) was induced with collagen type II (CII) in DBA/1 mice. The CD4^+^CD28^−^OX40^+^ T-cell subset and its cytokine production were detected by flow cytometry. After T-cell purification, adoptive transfer was performed in CIA mice. The regulatory role of OX40 was determined by blocking experiments in vitro and in vivo.

**Results:**

OX40 and OX40L were abnormally expressed in patients with RA and CIA mice. Further analysis showed that CD4^+^CD28^−^OX40^+^ T cells accumulated in patients with RA and in animal models. These cells produced higher levels of proinflammatory cytokines and were closely correlated with the clinicopathological features of the affected individuals. Adoptive transfer of CII-specific CD4^+^CD28^−^OX40^+^ T cells remarkably aggravated arthritic development and joint pathology in CIA mice. Moreover, OX40 blockade significantly reduced the proinflammatory responses and ameliorated arthritis development.

**Conclusions:**

OX40 acts as an alternative costimulator of CD4^+^CD28^−^ T cells and plays a pathogenic role in autoimmune arthritic development, suggesting that it is a potential target for immunomodulatory therapy of RA.

**Electronic supplementary material:**

The online version of this article (doi:10.1186/s13075-017-1261-9) contains supplementary material, which is available to authorized users.

## Background

Rheumatoid arthritis (RA) is a type of symmetric polyarticular arthritis, and T cell-mediated autoimmune responses to the articular synovium play a central role in the pathogenesis of RA [1, 2]. A marked accumulation of CD4^+^ T cells that lack CD28 expression is observed in patients with RA [3]. CD4^+^CD28^−^ T cells are autoreactive and could be regarded as effector memory T cells [4]. T-cell activation requires antigen recognition along with a second signal delivered by interactions between the costimulatory receptor on T cells and its ligands on antigen-presenting cells (APCs) [5]. Costimulatory molecules, including B7/CD28 and tumor necrosis factor (TNF)/tumor necrosis factor receptor (TNFR) superfamily members, are critical in antigen-specific T-cell responses. The absence of a second signal results in anergy and programmed cell death [6, 7]. However, loss of CD28 does not induce apoptosis in CD4^+^ T cells of patients with RA, suggesting that CD28-independent costimulatory pathways are involved in T-cell activation.

OX40 (CD134) and its ligand OX40L (CD252) are members of the TNF/TNFR superfamily. OX40 (TNFR superfamily member 4, TNFRSF4) is predominantly expressed by activated T cells, whereas the primary source of OX40L (TNF superfamily member 4, TNFSF4) is likely to be APCs. OX40 ligation augments the effector differentiation and the survival of memory T cells. The roles of OX40 and OX40L have been examined in most well-established autoimmune models [8, 9]. Although TNFSF4 polymorphisms were shown not to be linked with RA susceptibility [10], blockade of OX40/OX40L in collagen-induced arthritis (CIA) mice dramatically ameliorated disease severity [11, 12]. This indicates that OX40 signal plays a critical role in the development of autoimmune arthritis. As an enhancer of effector memory T cells, OX40 may not be an initiator, but may be an accelerator, in the pathogenesis of RA. Association of OX40 expression with arthritis development should be further investigated. Meanwhile, whether OX40 signal is involved in the activation of CD4^+^CD28^−^ T cells in RA should be clarified.

In view of this, potential correlations were analyzed between the CD4^+^CD28^−^OX40^+^ T-cell subset and clinicopathological characteristics of affected individuals among patients with RA and CIA mice. After cell sorting, the arthritis-associated pathogenic role of CD4^+^CD28^−^OX40^+^ T cells was investigated. Moreover, using OX40 blockade in vitro and in vivo, we revealed regulatory roles of OX40/OX40L signaling in autoimmune arthritis.

## Methods

### Human subjects

A total of 71 patients with RA diagnosed according to the 2010 rheumatoid arthritis classification criteria were recruited [13]. Additionally, 44 sex- and age-matched patients with osteoarthritis (OA) and 47 healthy volunteers healthy controls (HCs) were included as control subjects. All subjects were recruited from the Third Affiliated Hospital of Soochow University, Jiangsu, China. Disease activity was evaluated according to the 28-joint Disease Activity Score (DAS28). The level of disease activity could be interpreted as rheumatoid arthritis with low disease activity (Lo-RA), rheumatoid arthritis with moderate disease activity (Mo-RA), rheumatoid arthritis with high disease activity (Hi-RA), or rheumatoid arthritis with remission (Re-RA), according to the European League Against Rheumatism criteria [14]. Disease stages included early and late RA on the basis of criteria for early RA [15]. Nine patients with RA received methotrexate (MTX; Shanghai Sine Pharmaceutical Laboratories Co., Shanghai, China) therapy (10 mg/week for 20 weeks by oral administration). None of the patients had received immunosuppressive drugs within 1 year before the study period. Peripheral blood (PB) and synovial fluid (SF) samples were obtained from subjects after informed consent was provided according to a protocol approved by the local ethics committee of Soochow University. Sampling was completed in accordance with the guidelines of the local institutional review boards. Data about the patients and HCs are presented in supplementary figures and tables (Additional file 1: Table S1).

### CIA induction and corticosteroid treatment

Male DBA/1 mice at 8–10 weeks of age were obtained from the Shanghai Laboratory Animal Center and were maintained in a specific pathogen-free animal facility at Soochow University. All animal experiments conducted in this study were approved by the University Committee on the Use of Live Animals in Teaching and Research at our institution. Male DBA/1 mice were immunized intradermally at the base of the tail with 200 μg of bovine collagen type II (CII; Chondrex, Redmond, WA, USA) dissolved in 100 μl of 0.05 M acetic acid and mixed with an equal volume of complete Freund’s adjuvant containing heat-killed *Mycobacterium tuberculosis* (H37Ra strain, 4 mg/ml; Chondrex). Three weeks later, animals were reimmunized with 200 μg of CII emulsified in incomplete Freund’s adjuvant (Chondrex). Mice were scored for clinical signs as follows (per paw): 0, paws with no swelling; 1, paws with swelling of finger joints or focal redness; 2, paws with mild swelling of the wrist or ankle joints; 3, paws with severe swelling of the entire paw; and 4, paws with deformity or ankylosis. CIA mice were grouped into acute collagen-induced arthritis (A-CIA) and chronic collagen-induced arthritis (C-CIA) stages according to the criteria described by Thornton et al. [16]. On day 35 after the first immunization, dexamethasone (Dex; Tianjin Pharmaceutical Jiaozuo Co., Tianjin, China) was intraperitoneally injected for 7 days, including low dose (L-dose, 0.5 mg/kg/day), high dose (H-dose, 2 mg/kg/day), and a PBS control.

### Sample preparation and flow cytometry

For PB samples, the fluorochrome-labeled monoclonal antibodies (mAbs) antihuman CD4, CD28, and OX40 were added to 80 μl of whole blood before erythrocyte lysis was performed. Synovial fluid mononuclear cells (SFMCs) were isolated after SF samples were treated with hyaluronidase (10 μg/ml; Sigma-Aldrich, St. Louis, MO, USA). The mAbs described above were added to 100-μl SFMC suspensions (2 × 10^6^ cells/ml). For CIA mice, fluorochrome-labeled antimouse mAbs were added to 100-μl splenocyte suspensions (1 × 10^6^ cells/ml). Cells were incubated and evaluated using a COULTER EPICS XL flow cytometer (Beckman Coulter, Brea, CA, USA). The gating strategy for the CD4^+^CD28^−^OX40^+^ T-cell subset is described in supplementary figures and tables (Additional file 1: Fig. S1). For intracellular staining, peripheral blood mononuclear cells (PBMCs; 3 × 10^6^/well) were stimulated with phorbol 12-myristate 13-acetate (PMA, 50 ng/ml; eBioscience, San Diego, CA, USA) and ionomycin (1 μg/ml; eBioscience). Antihuman CD4, CD28, and OX40 mAbs were added before fixation and permeabilization, followed by the addition of phycoerythrin (PE)-cyanine 7 (Cy7)-conjugated antihuman interferon (IFN)-γ (clone 4S.B3; BioLegend, San Diego, CA, USA), interleukin (IL)-4 (clone MP4-25D2; BioLegend), or IL-17A (clone BL168; BioLegend) mAbs. Intracellular cytokine production was assessed using an FC 500 analyzer (Beckman Coulter). Information about all antibodies is provided in supplementary figures and tables (Additional file 1: Table S2).

### Adoptive transfer of CD4^+^CD28^−^OX40^+^ T cells

On day 28 after the second immunization, CIA mice were killed, and mononuclear splenocytes were stimulated in vitro with CII (30 μg/ml) for 72 h. CD4^+^ T cells were selected from mononuclear splenocytes using CD4^+^ microbeads (Miltenyi Biotec, Bergisch Gladbach, Germany) to over 97% purity. After CD28 and OX40 antimouse mAbs were added, T-cell subsets were sorted using a FACSAria™ II cell sorter (BD Biosciences, Franklin Lakes, NJ, USA). Sorted T cells (1 × 10^6^ cells in 200 μl of PBS) were injected intravenously into CIA mice on day 0 after the second immunization. After being fixed and decalcified, mouse ankles were embedded in paraffin and sectioned at 5-μm thickness before being stained with hematoxylin and eosin (H&E).

### OX40/OX40L blockage in vitro

On day 28 after the second immunization, mononuclear splenocytes (1 × 10^5^/well) of CIA mice were seeded onto 96-well tissue culture plates (Corning, Corning, NY, USA) in 10% FBS/RPMI 1640 medium and stimulated with CII (30 μg/ml) or anti-CD3 mAb (clone 145-2C11, 1 μg/ml; BioLegend) in the presence of antimouse OX40L mAb (clone RM134L; BioLegend). Rat immunoglobulin G (IgG) (clone RTK4530; BioLegend) was added as a control. After 72 h of incubation, cell proliferation was determined using a Cell Counting Kit-8 (CCK-8; Dojindo Laboratories, Mashikimachi, Japan). Supernatant cytokine levels were assessed using a mouse Th1/Th2/Th17 cytokine kit (BD Biosciences) according to the manufacturer’s instructions.

### OX40/OX40L blockade in vivo

On days 1–4 after the second immunization, randomized mice were injected intraperitoneally with antimouse OX40L mAb (100 μg/mouse/day) or IgG as controls. In another experiment, mice with similar arthritis scores were treated with anti-OX40L mAb or IgG on days 14–17 after the second immunization. On days 28 and 39 after anti-OX40L mAb early treatment, mononuclear splenocytes (1 × 10^5^/well) were stimulated with CII (30 μg/ml) for 72 h. Cell proliferation, supernatant cytokine levels, and intracellular staining were evaluated as described above. The PE-Cy7-conjugated antimouse mAbs used were as follows: IFN-γ (clone XMG1.2), IL-4 (clone 11B11), TNF-α (clone MP6-XT22), and IL-17A (clone TC11-18H10.1) (all from BioLegend).

### Statistical analysis

All statistical analyses were performed using IBM SPSS Statistics for Windows version 20.0 software (IBM, Armonk, NY, USA). All quantitative data are presented as the mean ± SD. Student’s *t* test or nonparametric Mann-Whitney *U* test was used for independent samples. A paired-samples *t* test or a nonparametric Wilcoxon signed-rank test was performed for paired samples. For multiple comparisons, one-way analysis of variance or the Kruskal-Wallis test was performed. A Spearman’s *r* value was calculated for correlation analyses. A *P* value less than 0.05 was considered to denote a significant difference.

## Results

### Abnormal expression of OX40 and OX40L in patients with RA and CIA mice

**Fig. 1:**
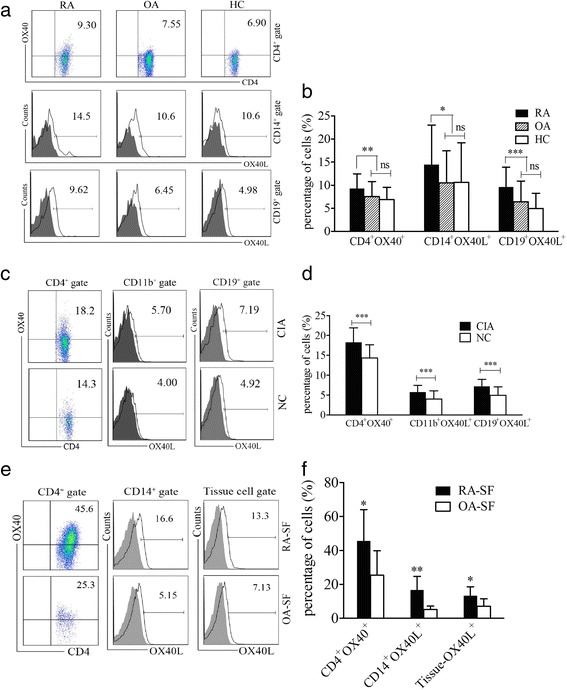
Expression of OX40 and its ligand OX40L in patients with RA and CIA mice. **a**, **c**, **e** Flow cytometry detection of OX40 and OX40L expression on immune cells in (**a**) PB or (**e**) SF samples of patients with RA, patients with OA, and HCs or (**c**) the spleen samples of CIA and NC mice. Data indicate the mean (%) of OX40^+^ or OX40L^+^ cells among gated cells. *Solid lines* indicate specific staining of OX40L, and *gray shading* represents isotype controls (scatter diagrams of isotype controls are not shown). **b** Percentages of CD4^+^OX40^+^, CD14^+^OX40L^+^, and CD19^+^OX40L^+^ cells in the PB samples of patients with RA (*n* = 71), patients with OA (*n* = 44), and HCs (*n* = 47). **d** Percentages of CD4^+^OX40^+^, CD11b^+^OX40L^+^, and CD19^+^OX40L^+^ cells in the spleen samples of CIA (*n* = 40) and NC (*n* = 30) mice. **f** Percentages of CD4^+^OX40^+^, CD14^+^OX40L^+^ and tissue OX40L^+^ cells in the SF samples of patients with RA (*n* = 10) and patients with OA (*n* = 10). Bars indicate mean ± SD; * *P* < 0.05, ** *P* < 0.01, *** *P* < 0.001, ns = not significant. *CIA* Collagen-induced arthritis, *HC* Healthy control subject, *NC* Normal control mice, *OA* Osteoarthritis, *PB* Peripheral blood, *RA* Rheumatoid arthritis, *SF* Synovial fluid

Flow cytometric analysis demonstrated that OX40 expression on CD4^+^ T cells in PB samples was more frequent in patients with RA than in patients with OA or HCs (9.30 ± 3.14% vs 7.55 ± 3.26%, 6.90 ± 2.64%, respectively, *P* = 0.008) (Fig. 1a and b). Expression of OX40L on CD14^+^ monocytes (*P* = 0.034) and CD19^+^ B cells (*P* < 0.001) also increased significantly in PB samples of patients with RA (Fig. 1a and b). In animal models, we found that OX40 and OX40L expression in spleen samples of CIA mice increased significantly compared with normal control mice (NC) (all *P* < 0.001) (Fig. 1c and d). Furthermore, SF samples exhibited increased expression of OX40 and OX40L in patients with RA (all *P* < 0.05) (Fig. 1e and f).

### Accumulation of CD4^+^CD28^−^OX40^+^ T cells in patients with RA and CIA mice

**Fig. 2:**
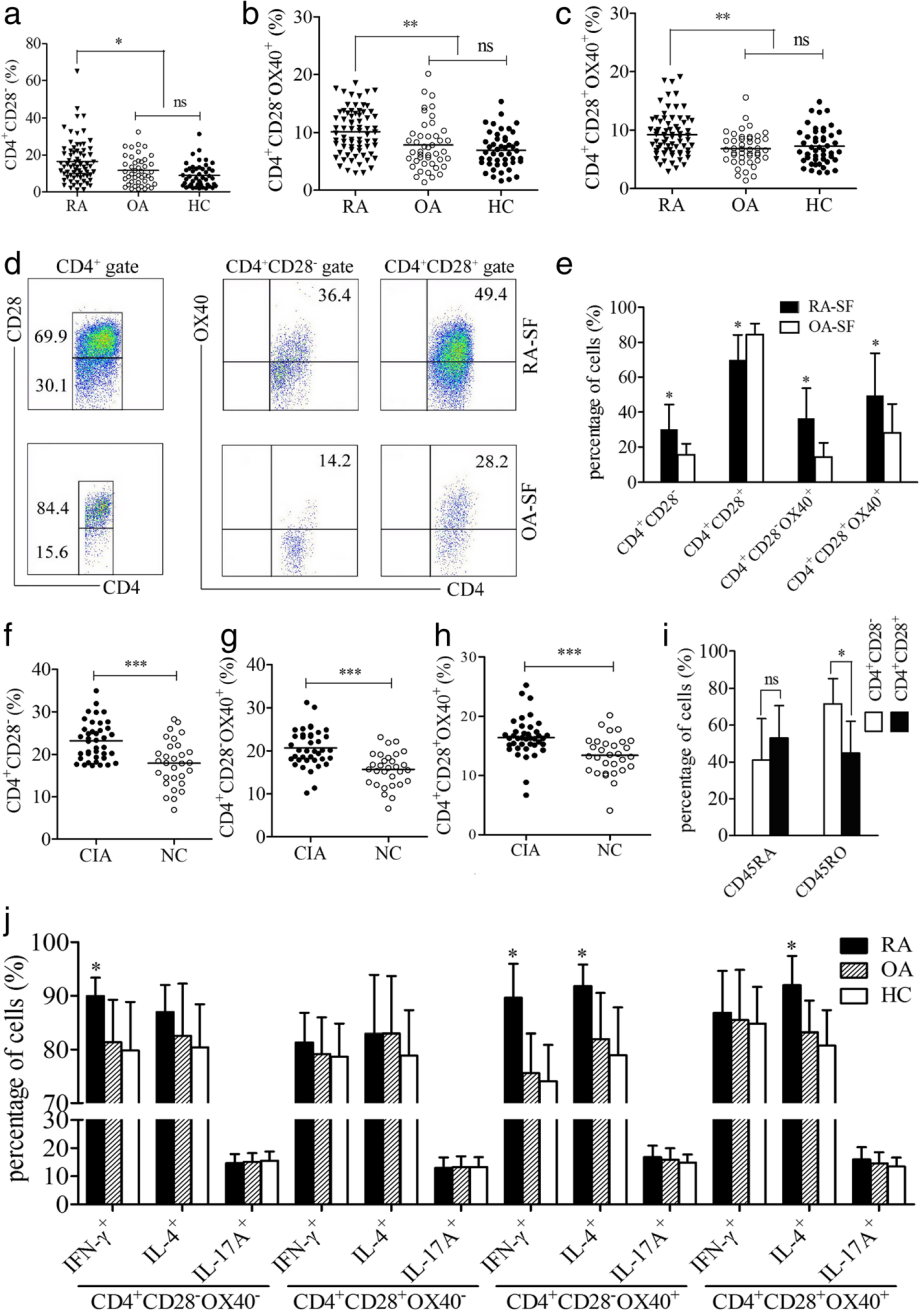
The CD4^+^CD28^−^OX40^+^ T-cell subset in patients with RA and CIA mice. **a** Percentages of CD4^+^CD28^−^ T cells in the PB samples of patients with RA (*n* = 71), patients with OA (*n* = 44), and HCs (*n* = 47). **b** Percentages of CD4^+^CD28^−^OX40^+^ T cells in the PB samples of patients with RA and control subjects. **c** Percentages of CD4^+^CD28^+^OX40^+^ T cells in the PB samples of patients with RA and control subjects. **d** Flow cytometric analyses of CD4^+^CD28^−^OX40^+^ T cells in SF samples of patients with RA and patients with OA (isotype controls not shown). Values indicate the mean (%) of T-cell subsets. **e** Percentages of CD4^+^CD28^−^, CD4^+^CD28^−^OX40^+^, and CD4^+^CD28^+^OX40^+^ T cells in the SF samples of patients with RA (*n* = 5) and patients with OA (*n* = 5). **f**, **g**, and **h** Percentages of CD4^+^CD28^−^ (**f**), CD4^+^CD28^−^OX40^+^ (**g**), and CD4^+^CD28^+^OX40^+^ (**h**) T cells in the spleen samples of CIA (*n* = 40) and NC (*n* = 30) mice. **i** Expression levels of CD45RA and CD45RO on CD4^+^CD28^−^ and CD4^+^CD28^+^ T cells in the PB samples of patients with RA (*n* = 5). **j** Cytokine production levels of CD4^+^CD28^−^OX40^+^ and the other T-cell subsets in PB samples of patients with RA (*n* = 5) and control subjects (*n* = 5). Each data point represents an individual subject; *horizontal lines* represent the mean; bars show mean ± SD; * *P* < 0.05, ** *P* < 0.01, *** *P* < 0 .001, ns = not significant. *CIA* Collagen-induced arthritis, *HC* Healthy control subject, *NC* Normal control mice, *OA* Osteoarthritis, *PB* Peripheral blood, *RA* Rheumatoid arthritis, *SF* Synovial fluid

Flow cytometric assessments of PB samples revealed a higher percentage of CD4^+^CD28^−^ T cells in patients with RA than in patients with OA and HCs (*P* = 0.021) (Fig. 2a). Further analysis showed that OX40 expression on CD4^+^CD28^−^ T cells was significantly increased in PB samples from patients with RA compared with those from control subjects (10.14 ± 3.98% vs 7.83 ± 4.28%, 6.91 ± 3.13%, respectively; *P* = 0.006) (Fig. 2b). Meanwhile, OX40 expression on CD4^+^CD28^+^ T cells was also elevated in PB samples of patients with RA compared with that in control subjects (*P* = 0.004) (Fig. 2c). Additionally, enrichment of CD4^+^CD28^−^, CD4^+^CD28^−^OX40^+^, and CD4^+^CD28^+^OX40^+^ T cells could be observed in SF samples from patients with RA (all *P* < 0.05) (Fig. 2d and e). However, the percentages of CD4^+^CD28^−^OX40^+^ and CD4^+^CD28^+^OX40^+^ T cells were comparable in both PB and SF samples of patients with RA (both *P* > 0.05). Similar to patients with RA, a higher percentage of CD4^+^CD28^−^, CD4^+^CD28^−^OX40^+^, and CD4^+^CD28^+^OX40^+^ T-cell subsets could be observed in spleen samples of CIA mice than in NCs (all *P* < 0.001) (Fig. 2f–h). Moreover, CD4^+^CD28^−^ T cells displayed enhanced CD45RO expression compared with CD4^+^CD28^+^ T cells in PB samples of patients with RA (Fig. 2i). Intracellular staining revealed that PMA/ionomycin-stimulated CD4^+^CD28^−^OX40^+^ T cells produced more IFN-γ (*P* = 0.026) and IL-4 (*P* = 0.042) in PB samples of patients with RA than those in control subjects. For CD4^+^CD28^−^OX40^−^ or CD4^+^CD28^+^OX40^+^ T cells, just IFN-γ (*P* = 0.036) or IL-4 (*P* = 0.044) secretion was increased in patients with RA, respectively. No significant difference was observed in cytokine production of CD4^+^CD28^+^OX40^−^ T cells between patients with RA and control subjects (Fig. 2j).

### CD4^+^CD28^−^OX40^+^ T-cell subsets closely correlate with clinicopathological characteristics of patients with RA and CIA mice

**Fig. 3:**
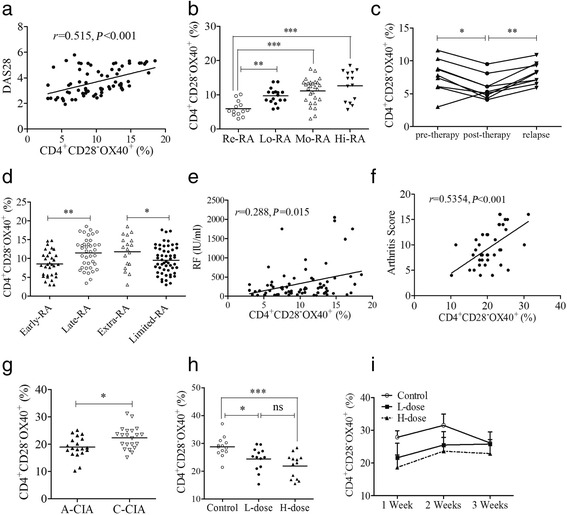
Correlations between CD4^+^CD28^−^OX40^+^ T cells and clinicopathological characteristics. **a** Correlation between DAS28 and the percentages of this subset (*n* = 71) in patients with RA. **b** Subset percentages of patients with Re-RA (*n* = 13), Lo-RA (*n* = 17), Mo-RA (*n* = 27), and Hi-RA (*n* = 14). **c** Changes of this subset in patients with RA treated with MTX (*n* = 9). **d** Percentages of this subset in patients with Early-RA (*n* = 32), Late-RA (*n* = 39), Extra-RA (*n* = 18), and Limited-RA (*n* = 53). **e** Correlation between RF levels and percentages of this subset (*n* = 71) in patients with RA. **f** Correlation between the arthritis scores of CIA mice and the percentages of this subset (*n* = 40). **g** Percentages of this subset in A-CIA (*n* = 19) and C-CIA (*n* = 21) mice. **h** Subset percentages in CIA mice treated with the L-dose (*n* = 13) or H-dose (*n* = 13) of Dex or PBS (*n* = 13). **i** Changes of this subset after Dex treatment. Each data point represents an individual subject; *horizontal lines* represent means; the *r* value indicates Spearman’s correlation coefficient; * *P* < 0.05, ** *P* < 0.01, *** *P* < 0.001, ns = not significant. *A-CIA* Acute collagen-induced arthritis, *CIA* Collagen-induced arthritis, *C-CIA* Chronic collagen-induced arthritis, *Dex* Dexamethasone, *Extra-RA* Rheumatoid arthritis with extraarticular manifestations, *H-dose* High dose, *Hi-RA* Rheumatoid arthritis with high disease activity, *L-dose* Low dose, *Limited-RA* Rheumatoid arthritis with limited joint manifestations, *Lo-RA* Rheumatoid arthritis with low disease activity, *Mo-RA* Rheumatoid arthritis with moderate disease activity, *MTX* Methotrexate, *RA* Rheumatoid arthritis, *Re-RA* Rheumatoid arthritis with remission, *RF* Rheumatoid factor

The DAS28 was used to evaluate arthritic activity, which was positively correlated with the percentage of CD4^+^CD28^−^OX40^+^ T cells in PB samples of patients with RA (*r* = 0.515, *P* < 0.001) (Fig. 3a). Further analysis showed significant differences among the percentages of CD4^+^CD28^−^OX40^+^ T cells in PB samples of patients with Re-RA (5.94 ± 2.24%), Lo-RA (9.68 ± 2.31%, *P* = 0.001), Mo-RA (11.14 ± 3.12%, *P* < 0.001), and Hi-RA (12.65 ± 4.47%, *P* < 0.001) (all vs Re-RA) (Fig. 3b). MTX significantly relieved arthritis activity and reduced the percentage of CD4^+^CD28^−^OX40^+^ T cells in PB samples of patients with RA (5.93 ± 1.80% vs 7.74 ± 2.54% posttherapy vs pretherapy, *P* = 0.038) after 16 weeks of treatment. However, follow-up in the 32nd week showed that, with arthritis relapse, the percentage of this T-cell subset was higher than in the 16th week (8.10 ± 1.62% vs 5.93 ± 1.80%, *P* = 0.008) (Fig. 3c). In different clinical stages, patients with Late-RA exhibited a higher percentage of CD4^+^CD28^−^OX40^+^ T cells in PB samples than patients with Early-RA (*P* = 0.002) (Fig. 3d, *left*). Additionally, there was a significant difference in the percentage of this subset in PB samples between patients with rheumatoid arthritis with extraarticular manifestations (Extra-RA) and patients with rheumatoid arthritis with limited joint manifestations (Limited-RA) (*P* = 0.036) (Fig. 3d, *right*). As an important clinical indicator, the levels of rheumatoid factor (RF) were positively correlated with the percentage of CD4^+^CD28^−^OX40^+^ T cells in PB samples of patients with RA (*r* = 0.288, *P* = 0.015) (Fig. 3e). In spleen samples of animal models, a positive correlation was found between arthritis scores and the percentages of the CD4^+^CD28^−^OX40^+^ T-cell subset (*r* = 0.5354, *P* = 0.0004) (Fig. 3f). During different phases of disease, the number of CD4^+^CD28^−^OX40^+^ T cells was increased in spleen samples of C-CIA mice compared with that in A-CIA mice (Fig. 3 g). Both L-dose and H-dose Dex markedly alleviated arthritic development in CIA mice (supplementary figures and tables in Additional file 1: Fig. S2) and reduced the percentages of CD4^+^CD28^−^OX40^+^ T cells in spleen samples (Fig. 3 h). Three weeks after low doses of Dex administration, the percentage of CD4^+^CD28^−^OX40^+^ T cells in spleen samples of CIA mice approximated that of the control levels (Fig. 3i).

### Adoptive transfer of CD4^+^CD28^−^OX40^+^ T cells aggravated arthritic progression in CIA mice

**Fig. 4:**
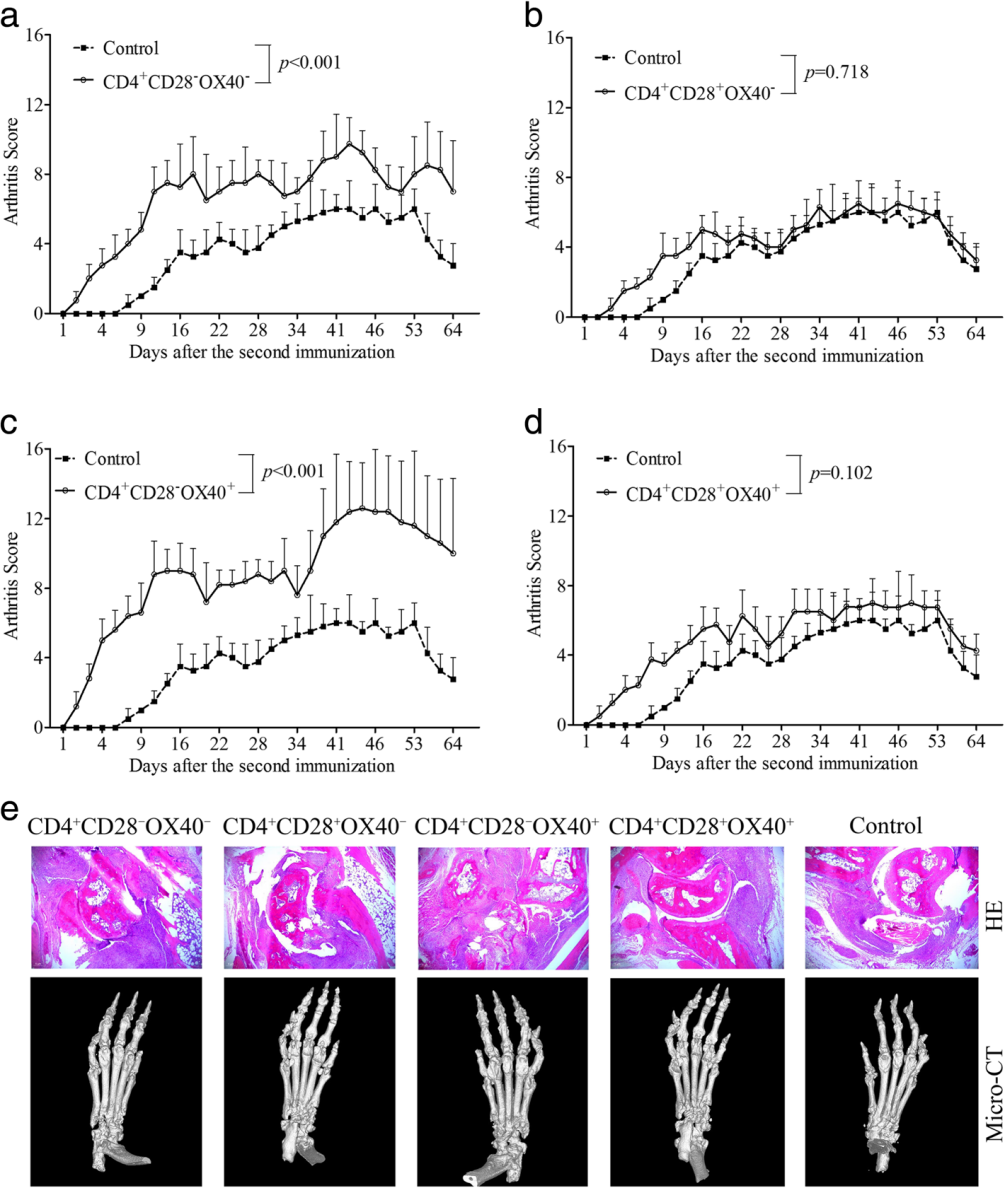
Adoptive transfer in CIA mice. Passive CIA was induced by intravenous transfer of sorted CD4^+^CD28^−^OX40^−^ (**a**, *n* = 4), CD4^+^CD28^+^OX40^−^ (**b**, *n* = 4), CD4^+^CD28^−^OX40^+^ (**c**, *n* = 5), or CD4^+^CD28^+^OX40^+^ (**d**, *n* = 4) T-cell subsets. CIA mice that received transfers with CD4^+^CD28^−^OX40^−^ and CD4^+^CD28^−^OX40^+^ T cells had aggravated arthritis development compared with control CIA mice (*n* = 4). CD4^+^CD28^+^OX40^−^ and CD4^+^CD28^+^OX40^+^ T cells did not significantly affect arthritis development compared with control CIA mice. The arthritis scores of different groups of recipient mice are shown as the mean ± SD. **e** H&E histological stains (×200 original magnification) and micro-computed tomographic analysis of representative ankle sections from the experiments shown in **a**, **b**, **c**, and **d**. *CIA* Collagen-induced arthritis, *H&E* Hematoxylin and eosin

Arthritic onset in CIA mice that received transfer with CD4^+^CD28^−^OX40^+^ T cells occurred much earlier, and the arthritis scores in these mice were much higher than those in control CIA mice (8.55 ± 3.17 vs 3.48 ± 2.12, *P* < 0.001) (Fig. 4c). Meanwhile, arthritis onset and scores in CIA mice that received transfer with CD4^+^CD28^−^OX40^−^ T cells also showed a significant difference from those in control CIA mice (6.57 ± 2.47 vs 3.48 ± 2.12, *P* < 0.001) (Fig. 4a). Although CD4^+^CD28^+^OX40^−^ or CD4^+^CD28^+^OX40^+^ T-cell transfer slightly accelerated early arthritic development in CIA mice, these scores did not reach significance compared with control CIA mice (*P* = 0.718 and *P* = 0.102, respectively) (Fig. 4b and d). Furthermore, among the four T-cell subsets transferred to CIA mice, CD4^+^CD28^−^OX40^+^ T cells displayed the most significant pathogenic role in arthritis development compared with CD4^+^CD28^−^OX40^−^ (*P* = 0.011), CD4^+^CD28^+^OX40^−^ (*P* < 0.001), and CD4^+^CD28^+^OX40^+^ (*P* < 0.001) T cells. Severe synovial and periarticular inflammation with abnormal narrowing of the joint space was observed in H&E staining of CIA mice transferred with CD4^+^CD28^−^OX40^+^ T cells. Similarly, micro-computed tomography revealed the most serious bone erosion in CIA mice that received transfers with CD4^+^CD28^−^OX40^+^ T cells compared with those in control CIA mice (Fig. 4e).

### OX40 blockade significantly inhibited proinflammatory responses in vitro

**Fig. 5:**
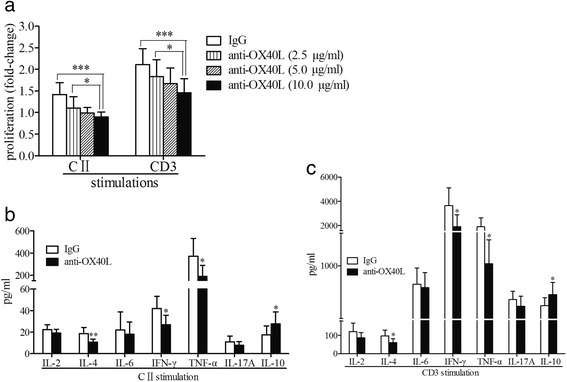
OX40/OX40L blockade in vitro. **a** Cell proliferation in the CII- or CD3-stimulated splenocytes of CIA mice in the presence of anti-OX40L mAb (2.5 μg/ml, *n* = 19; 5.0 μg/ml, *n* = 19; 10.0 μg/ml, *n* = 19) or IgG controls (*n* = 19). **b**, **c** Cytokine secretion in the supernatants of CII-stimulated (**b**) or CD3-stimulated (**c**) splenocytes from CIA mice in the presence of anti-OX40L mAb (5.0 μg/ml, *n* = 8) or in IgG controls (*n* = 8). Bars show means ± SD; * *P* < 0.05, ** *P* < 0.01, *** *P* < 0.001. *CIA* Collagen-induced arthritis, *CII* Collagen type II, *IgG* Immunoglobulin G, *mAb* Monoclonal antibody

In splenocytes stimulated with CII, OX40L-blocking antibodies at 2.5 μg/ml (*P* = 0.006), 5.0 μg/ml (*P* < 0.001), and 10.0 μg/ml (*P* < 0.001) displayed inhibitory effects on cell proliferation compared with IgG controls (Fig. 5a, *left*). Simultaneously, antibodies at 5.0 μg/ml (*P* = 0.002) and 10.0 μg/ml (*P* < 0.001) exhibited depressing effects on the proliferation of splenocytes activated by CD3 stimuli (Fig. 5a, *right*). Further studies showed that applying the blocking antibody at 5.0 μg/ml remarkably reduced the cytokine secretion of CII-stimulated splenocytes, including IFN-γ (26.86 ± 8.88 pg/ml vs 41.96 ± 11.23 pg/ml, *P* = 0.021), IL-4 (10.65 ± 2.85 pg/ml vs 18.67 ± 5.75 pg/ml, *P* = 0.005), and TNF-α (190.59 ± 90.11 pg/ml vs 371.86 ± 160.72 pg/ml, *P* = 0.021) (all vs IgG controls). However, levels of IL-10 were elevated (*P* = 0.046), and the secretion of IL-2, IL-6, and IL-17A were not significantly different from those in the IgG controls (all *P* > 0.05) (Fig. 5b). Additionally, OX40 blockade showed similar effects on cytokine production in splenocytes stimulated with CD3 (Fig. 5c).

### OX40 blockade ameliorated arthritic progression in CIA mice

**Fig. 6:**
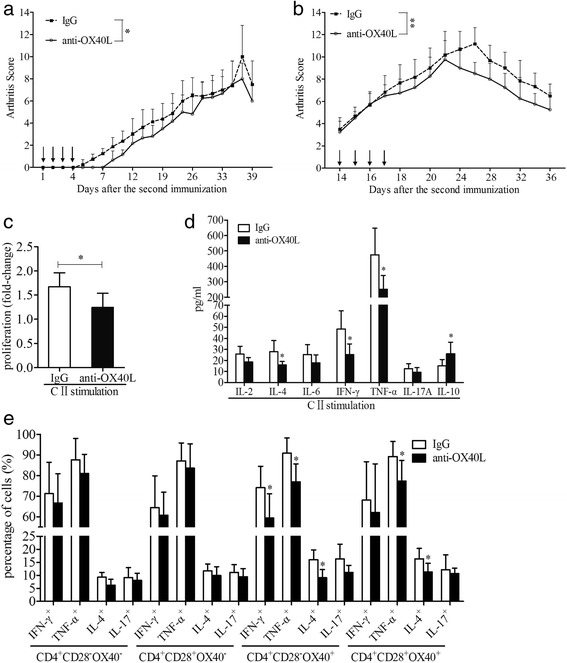
OX40/OX40L blockade in vivo. **a**, **b** Treatment with anti-OX40L mAb on days 1–4 (**a**, *n* = 6) (IgG, *n* = 8) and days 14–17 (**b**, *n* = 4) (IgG, *n* = 6) after the second immunization ameliorated arthritis development in CIA mice. *Arrows* indicate the days of dosing. **c** Splenocytes from CIA mice treated with anti-OX40L mAb (*n* = 6) demonstrated reduced proliferation in CII stimulation compared with those treated with IgG (*n* = 6). **d** Cytokine secretion in the supernatants of splenocytes from CIA mice treated with anti-OX40L mAb (*n* = 6) or IgG controls (*n* = 6). **e** Cytokine production of CD4^+^CD28^−^OX40^+^ and the other control T-cell subsets after the splenocytes from CIA mice treated with anti-OX40L mAb (*n* = 5) or IgG controls (*n* = 5) were stimulated with PMA/ionomycin. Bars show mean ± SD; * *P* < 0.05, ** *P* < 0.01. *CIA* Collagen-induced arthritis, *CII* Collagen type II, *IgG* Immunoglobulin G, *mAb* Monoclonal antibody

Early treatment starting on day 1 after the second immunization indicated that OX40 blockade in vivo delayed arthritis onset and reduced the arthritis scores of CIA mice compared with IgG controls (*P* = 0.013) (Fig. 6a). Correspondingly, late treatment starting on day 14 after the second immunization also evidently alleviated arthritis development (*P* = 0.008) (Fig. 6b). Further studies demonstrated that OX40 blockade in vivo reduced cell proliferation (*P* = 0.037) (Fig. 6c) and cytokine secretion, including IFN-γ (*P* = 0.025), IL-4 (*P* = 0.025), and TNF-α (*P* = 0.037), by CII-stimulated splenocytes (Fig. 6d). Accordingly, in vivo changes in IL-10, IL-2, IL-6, and IL-17A levels were consistent with the in vitro experiment (Fig. 6d). Intracellular staining showed that OX40 blockade in vivo significantly suppressed cytokine secretion by CD4^+^CD28^−^OX40^+^ T cells, including IFN-γ (59.38 ± 11.78% vs 74.10 ± 10.39%, *P* = 0.028), TNF-α (76.88 ± 8.77% vs 90.84 ± 7.43%, *P* = 0.028), and IL-4 (9.11 ± 3.12% vs 16.01 ± 3.89%, *P* = 0.016) (all vs IgG controls). However, the levels of IL-17A were not significantly different (Fig. 6e). For CD4^+^CD28^+^OX40^+^ T cells, TNF-α and IL-4 were reduced by OX40 blockade (both *P* < 0.05). With regard to CD4^+^CD28^−^OX40^−^ and CD4^+^CD28^+^OX40^−^ T cells, OX40 blockade had no impact on the secretion of IFN-γ, TNF-α, IL-4, and IL-17A compared with IgG controls (all *P* > 0.05) (Fig. 6e).

## Discussion

Recently, the self-activation mechanisms of CD4^+^CD28^−^ T cells have been explored, including allelic associations [17], cytokine production [18, 19], cell receptor stimulation [20, 21], and DNA methylation [22, 23]. However, in the absence of CD28, which costimulatory molecules mediate the second signal is not yet well known. Researchers in a recent study reported that OX40 expression was upregulated on CD4^+^CD28^−^ T cells in patients with acute coronary syndrome and that the secretion of IFN-γ and TNF-α was suppressed by OX40 blockade [24]. However, whether abnormal OX40 signaling is involved in the self-responses of CD4^+^CD28^−^ T cells in patients with RA remains unclear. In this study, a CD4^+^CD28^−^OX40^+^ T-cell subset was established to be clinicopathologically significant, and its autoreactivation and pathogenicity were demonstrated in patients with RA and CIA mice. Additionally, the regulatory roles of OX40 signaling in CD4^+^CD28^−^ T cells were verified in vitro and in vivo.

In accord with previous studies [25], abnormal expression of OX40 in patients with RA and CIA mice suggested that an OX40 costimulatory signal was involved in the pathological development of autoimmune arthritis. Further upregulated expression of OX40 in SF of patients with RA implied that OX40 signal may promote abnormal activation of autoreactive T cells after migration of CD4^+^ T into the inflammatory microenvironment. In patients with RA, enhanced OX40 expression was observed not only on CD4^+^CD28^−^ but also on CD4^+^CD28^+^ T cells. OX40 expression levels did not depend on CD28 expression in RA, suggesting that OX40 costimulated T cells in a CD28-independent way. In addition, CD4^+^CD28^−^ T cells with enhanced CD45RO expression suggested the characteristics of memory T cells, and this population is enriched in patients with RA, probably because of OX40 overexpression. Elevated levels of IFN-γ and IL-4 suggest that CD4^+^CD28^−^OX40^+^ T cells may be involved in Th1/Th2 responses and help to produce autoantibody, such as RF [26]. However, CD4^+^CD28^−^OX40^−^ may just function in a Th1 pathway, and for CD4^+^CD28^+^OX40^+^ T cells, a Th2 pathway may be involved. These results implied that CD4^+^CD28^−^OX40^+^ T cells were distinct from the other T-cell subsets and may exhibit specific characteristics in autoimmune arthritis. Upon OX40 costimulation, effector differentiation and long survival may emerge in memory CD4^+^CD28^−^ T cells, which may increase disease activity and lead to a protracted course of disease, as described in another study [25]. As a consequence of OX40 overexpression, CD4^+^CD28^−^ T cells may differentiate diversely in response to multiple autoantigens, suggesting extraarticular manifestations, as mentioned in previous studies [3, 27]. Immunosuppressive agents regulate immune responses by changing the expression and function of costimulatory molecules [28, 29], and this regulatory role could be effectively reversed by OX40-mediated costimulatory signals [30]. Reduced OX40 signals by MTX or Dex treatment may suppress the autoactivation of CD4^+^CD28^−^ T cells and lead to arthritis remission. However, arthritis relapses after medicine withdrawal suggest that new specific approaches for therapy must be explored.

In adoptive transfer assays, although similar arthritis scores were observed on day 34 in the duration curves, CD4^+^CD28^−^OX40^+^ T cells demonstrated a more powerful pathogenic role than the CD4^+^CD28^−^OX40^−^, CD4^+^CD28^+^OX40^−^, and CD4^+^CD28^+^OX40^+^ T cells in both early (days 1–33) and late (days 35–64) arthritis. This finding indicates that, in early arthritis, activation of CD4^+^CD28^−^OX40^+^ T cells occurs in a short-lived effector- and autoantigen-dependent manner but probably acts in a long-lived memory- and autoantigen-independent fashion in late arthritis, in agreement with the OX40 properties described by Croft [8, 9]. Probably owing to natural killer cell or chemokine receptors expressed on CD4^+^CD28^−^ T cells [20, 21], mice that received transfers with CD4^+^CD28^−^OX40^−^ T cells exhibited deteriorated arthritis progression, especially in early arthritis. On one hand, this meant that, in early arthritis, additional mechanisms were involved in activation of CD4^+^CD28^−^ T cells, except for OX40 costimulation, On the other hand, long-lived memory characteristics of OX40 [8, 9] may take effects in late arthritis. Autoantigen-specific CD4^+^CD28^−^OX40^+^ T cells resulted in persistent autoimmune injuries and protracted joint damage. However, the absence of OX40 markedly reduced the survival of memory T cells, and late arthritis development was alleviated in the mice that received transfers with CD4^+^CD28^+^OX40^−^ T cells. Recent studies showed that OX40 costimulatory signals promoted differentiation of effector T lymphocytes in a CD28-independent way [31] and that “superstimulation” of combined CD28 and OX40 did not outperform CD28 by itself [32]. Additionally, the presence of OX40 may neutralize costimulation of CD28 [33], which may impair arthritis pathogenic roles of CD4^+^CD28^+^OX40^+^ T cells. A distinct pathogenic role has been further confirmed on the basis of different characteristics of CD28^−^ and CD28^+^ T cells [4]. The robust pathogenicity of CD4^+^CD28^−^ T cells may reflect OX40 characteristics of costimulating memory T cells in a CD28-independent manner, as described in a report by Demirci et al. [34].

In vitro different time points resulted in findings inconsistent with those of Yoshioka et al. [11], probably because of the diverse roles of OX40 in short-lived effector and long-lived memory cells [8, 9]. In our study, the blockade time point was day 28 after the second immunization, whereas in the study by Yoshioka et al., it was day 7. On day 7, OX40^+^ T cells may have occurred in the absence of short-lived effector responses, whereas OX40 memory characteristics may be generated on days 14–28. In vitro OX40 blockade on day 7 may have little effect. On the basis of in vitro studies, early treatment on days 1–4 in vivo showed results similar to those of the studies by Yoshioka et al. [11] and Gwyer Findlay et al. [12], probably owing to suppression of short-lived effector responses of OX40^+^ T cells. However, inconsistent with the findings of Yoshioka et al. [11], delayed treatment on days 14–17 also distinctly ameliorated arthritis symptoms, mainly owing to the different time points. On day 14 (blockade time point in our study), generation of memory T cells may be reduced by OX40 blockage, whereas short-lived effector responses resulting from OX40 may have faded on day 7 (blockade time point in study by Yoshioka et al.). Changes in cytokine levels further corroborated the findings in analysis of human samples. In the immunopathogenesis of RA, Th1 and Th2 responses are involved in different phases [2]. As observed in transfer assays, Th1/Th2-polarized CD4^+^CD28^−^OX40^+^ T cells played central roles in all phases of autoimmune arthritis. However, CD4^+^CD28^+^OX40^−^ and CD4^+^CD28^+^OX40^+^ T cells contributed to outcomes only in the early phases of the pathological process. For CD4^+^CD28^−^OX40^−^ T cells, although early Th1 response aggravated arthritic development in an OX40-independent way, progression faded in late-phase arthritis.

## Conclusions

A novel CD4^+^CD28^−^OX40^+^ T-cell subset was established to be clinicopathologically significant, suggesting that OX40 may act as a promising biomarker for RA. OX40 was characterized as an alternative costimulator for autoactivation of CD4^+^CD28^−^ T cells in autoimmune arthritis, and OX40 blockade may represent an effective approach for immunomodulatory therapy.

## Additional file

Additional file 1: Supplementary figures and tables. (DOC 614 kb)

## Abbreviations

A-CIA: Acute collagen-induced arthritis
APC: Antigen-presenting cell
CCK-8: Cell Counting Kit-8
CIA: Collagen-induced arthritis
C-CIA: Chronic collagen-induced arthritis
CII: Collagen type II
Cy7: Cyanine 7
DAS28: 28-joint Disease Activity Score
Dex: Dexamethasone
EULAR: European League Against Rheumatism
Extra-RA: Rheumatoid arthritis with extraarticular manifestations
H&E: Hematoxylin and eosin
HC: Healthy control subject
H-dose: High dose
Hi-RA: Rheumatoid arthritis with high disease activity
IFN: Interferon
IgG: Immunoglobulin G
IL: Interleukin
L-dose: Low dose
Limited-RA: Rheumatoid arthritis with limited joint manifestations
Lo-RA: Rheumatoid arthritis with low disease activity
mAb: Monoclonal antibody
Mo-RA: Rheumatoid arthritis with moderate disease activity
MTX: Methotrexate
NC: Normal control mice
OA: Osteoarthritis
PB: Peripheral blood
PBMC: Peripheral blood mononuclear cell
PE: Phycoerythrin
PMA: Phorbol 12-myristate 13-acetate
RA: Rheumatoid arthritis
Re-RA: Rheumatoid arthritis with remission
RF: Rheumatoid factor
SF: Synovial fluid
SFMC: Synovial fluid mononuclear cell
TNF: Tumor necrosis factor
TNFR: Tumor necrosis factor receptor
TNFRSF4: Tumor necrosis factor receptor superfamily member 4
TNFSF4: Tumor necrosis factor superfamily member 4

## References

[1] Scott DL, Wolfe F, Huizinga TW (2010). Rheumatoid arthritis. Lancet.

[2] Scrivo R, Di Franco M, Spadaro A, Valesini G (2007). The immunology of rheumatoid arthritis. Ann N Y Acad Sci.

[3] Pawlik A, Ostanek L, Brzosko I, Brzosko M, Masiuk M, Machalinski B (2003). The expansion of CD4^+^CD28^−^ T cells in patients with rheumatoid arthritis. Arthritis Res Ther.

[4] Pieper J, Johansson S, Snir O, Linton L, Rieck M, Buckner JH (2014). Peripheral and site-specific CD4^+^CD28^null^ T cells from rheumatoid arthritis patients show distinct characteristics. Scand J Immunol.

[5] Davis MM, Bjorkman PJ (1988). T cell antigen receptor genes and T cell recognition. Nature.

[6] Bour-Jordan H, Esensten JH, Martinez-Llordella M, Penaranda C, Stumpf M, Bluestone JA (2011). Intrinsic and extrinsic control of peripheral T-cell tolerance by costimulatory molecules of the CD28/B7 family. Immunol Rev.

[7] Watts TH (2005). TNF/TNFR family members in costimulation of T cell responses. Annu Rev Immunol.

[8] Redmond WL, Ruby CE, Weinberg AD (2009). The role of OX40-mediated co-stimulation in T-cell activation and survival. Crit Rev Immunol.

[9] Croft M (2010). Control of immunity by the TNFR-related molecule OX40 (CD134). Annu Rev Immunol.

[10] Lu MM, Xu WD, Yang J, Ye QL, Feng CC, Li J (2013). Association of TNFSF4 polymorphisms with systemic lupus erythematosus: a meta-analysis. Mod Rheumatol.

[11] Yoshioka T, Nakajima A, Akiba H, Ishiwata T, Asano G, Yoshino S (2000). Contribution of OX40/OX40 ligand interaction to the pathogenesis of rheumatoid arthritis. Eur J Immunol.

[12] Gwyer Findlay E, Danks L, Madden J, Cavanagh MM, McNamee K, McCann F (2014). OX40L blockade is therapeutic in arthritis, despite promoting osteoclastogenesis. Proc Natl Acad Sci U S A.

[13] Aletaha D, Neogi T, Silman AJ, Funovits J, Felson DT, Bingham CO (2010). 2010 Rheumatoid arthritis classification criteria: an American College of Rheumatology/European League Against Rheumatism collaborative initiative. Arthritis Rheum.

[14] Fransen J, van Riel PL (2009). The Disease Activity Score and the EULAR response criteria. Rheum Dis Clin North Am.

[15] Huizinga TW, Machold KP, Breedveld FC, Lipsky PE, Smolen JS (2002). Criteria for early rheumatoid arthritis: from Bayes’ law revisited to new thoughts on pathogenesis. Arthritis Rheum.

[16] Thornton S, Boivin GP, Kim KN, Finkelman FD, Hirsch R (2000). Heterogeneous effects of IL-2 on collagen-induced arthritis. J Immunol.

[17] Sun W, Cui Y, Zhen L, Huang L (2011). Association between HLA-DRB1, HLA-DRQB1 alleles, and CD4^+^CD28^null^ T cells in a Chinese population with coronary heart disease. Mol Biol Rep.

[18] Alonso-Arias R, Moro-García MA, Vidal-Castiñeira JR, Solano-Jaurrieta JJ, Suárez-García FM, Coto E (2011). IL-15 preferentially enhances functional properties and antigen-specific responses of CD4^+^CD28^null^ compared to CD4^+^CD28^+^ T cells. Aging Cell.

[19] Broux B, Mizee MR, Vanheusden M, van der Pol S, van Horssen J, Van Wijmeersch B (2015). IL-15 amplifies the pathogenic properties of CD4^+^CD28^−^ T cells in multiple sclerosis. J Immunol.

[20] Fasth AE, Björkström NK, Anthoni M, Malmberg KJ, Malmström V (2010). Activating NK-cell receptors co-stimulate CD4^+^CD28^−^ T cells in patients with rheumatoid arthritis. Eur J Immunol.

[21] Broux B, Mizee MR, Vanheusden M, van der Pol S, van Horssen J, Van Wijmeersch B (2012). CX_3_CR1 drives cytotoxic CD4^+^CD28^−^ T cells into the brain of multiple sclerosis patients. J Autoimmun.

[22] Chen Y, Gorelik GJ, Strickland FM, Richardson BC (2010). Decreased ERK and JNK signaling contribute to gene overexpression in “senescent” CD4^+^CD28^−^ T cells through epigenetic mechanisms. J Leukoc Biol.

[23] Liu Y, Chen Y, Richardson B (2009). Decreased DNA methyltransferase levels contribute to abnormal gene expression in “senescent” CD4^+^CD28^−^ T cells. Clin Immunol.

[24] Dumitriu IE, Baruah P, Finlayson CJ, Loftus IM, Antunes RF, Lim P (2012). High levels of costimulatory receptors OX40 and 4-1BB characterize CD4^+^CD28^null^ T cells in patients with acute coronary syndrome. Circ Res.

[25] Giacomelli R, Passacantando A, Perricone R, Parzanese I, Rascente M, Minisola G (2001). T lymphocytes in the synovial fluid of patients with active rheumatoid arthritis display CD134-OX40 surface antigen. Clin Exp Rheumatol.

[26] Knijff-Dutmer E, Drossaers-Bakker W, Verhoeven A, van der Sluijs VG, Boers M, van der Linden S (2002). Rheumatoid factor measured by fluoroimmunoassay: a responsive measure of rheumatoid arthritis disease activity that is associated with joint damage. Ann Rheum Dis.

[27] Fasth AE, Snir O, Johansson AA, Nordmark B, Rahbar A, Af Klint E (2007). Skewed distribution of proinflammatory CD4^+^CD28^null^ T cells in rheumatoid arthritis. Arthritis Res Ther.

[28] Duperrier K, Velten FW, Bohlender J, Demory A, Metharom P, Goerdt S (2005). Immunosuppressive agents mediate reduced allostimulatory properties of myeloid-derived dendritic cells despite induction of divergent molecular phenotypes. Mol Immunol.

[29] Barrat FJ, Cua DJ, Boonstra A, Richards DF, Crain C, Savelkoul HF (2002). In vitro generation of interleukin 10-producing regulatory CD4^+^ T cells is induced by immunosuppressive drugs and inhibited by T helper type 1 (Th1)- and Th2-inducing cytokines. J Exp Med.

[30] Ito T, Wang YH, Duramad O, Hanabuchi S, Perng OA, Gilliet M (2006). OX40 ligand shuts down IL-10-producing regulatory T cells. Proc Natl Acad Sci U S A.

[31] Williams CA, Murray SE, Weinberg AD, Parker DC (2007). OX40-mediated differentiation to effector function requires IL-2 receptor signaling but not CD28, CD40, IL-12Rβ2, or T-bet. J Immunol.

[32] Hombach AA, Rappl G, Abken H (2013). Arming cytokine-induced killer cells with chimeric antigen receptors: CD28 outperforms combined CD28^−^OX40 “super-stimulation.”. Mol Ther.

[33] Hombach AA, Heiders J, Foppe M, Chmielewski M, Abken H (2012). OX40 costimulation by a chimeric antigen receptor abrogates CD28 and IL-2 induced IL-10 secretion by redirected CD4^+^ T cells. Oncoimmunology.

[34] Demirci G, Amanullah F, Kewalaramani R, Yagita H, Strom TB, Sayegh MH (2004). Critical role of OX40 in CD28 and CD154-independent rejection. J Immunol.

