# Supplementary material for: OX40 signaling is involved in the autoactivation of CD4+CD28− T cells and contributes to the pathogenesis of autoimmune arthritis
Source: Arthritis Res Ther. 2017 Mar 21;19:67. doi: 10.1186/s13075-017-1261-9 (PMC5359925; doi:10.1186/s13075-017-1261-9)
Supplement: Additional file 1: — Supplementary figures and tables. (DOC 614 kb) [file 13075_2017_1261_MOESM1_ESM.doc]

**Supplementary figures and tables**


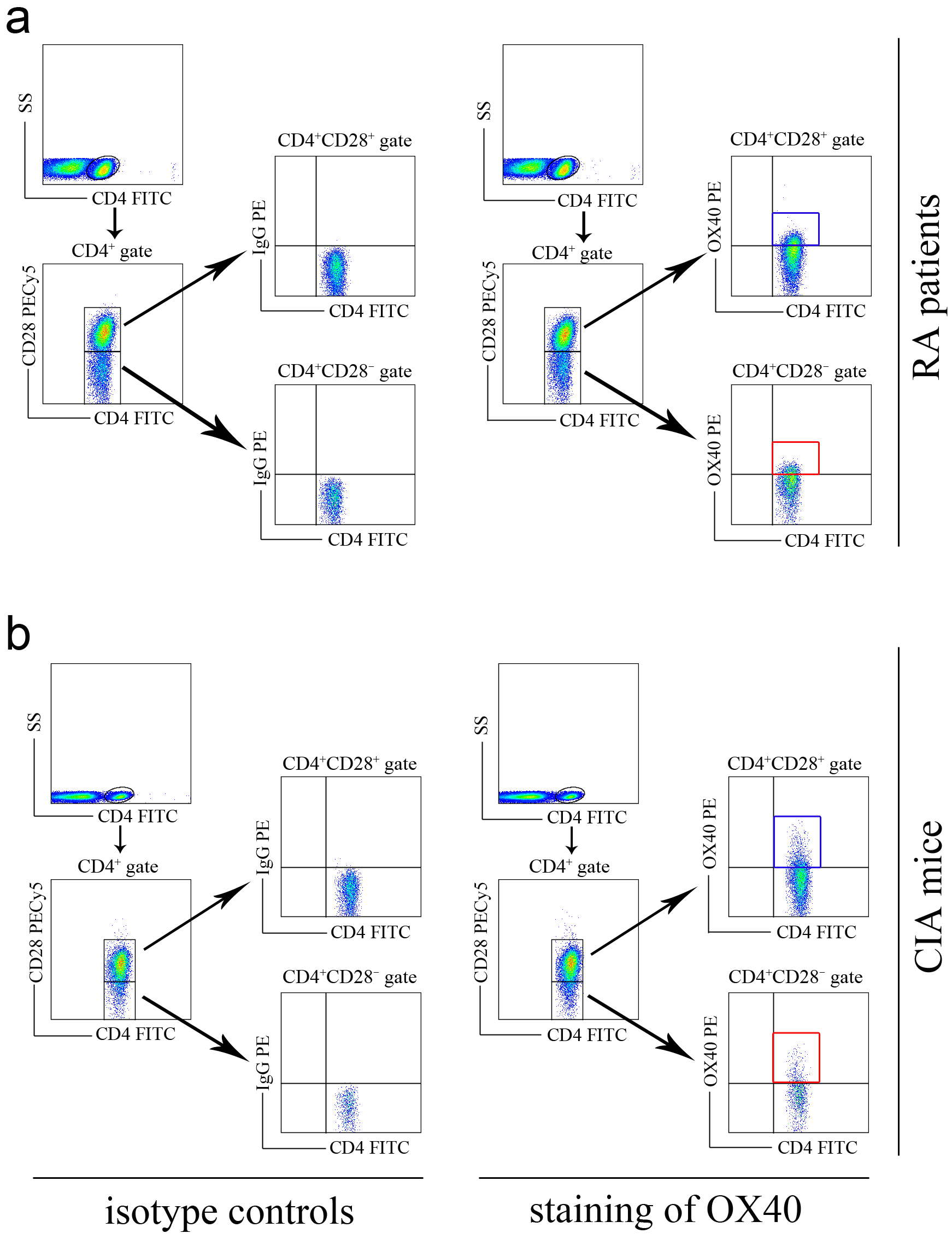


**Fig. S1.** Gating strategy for CD4^+^CD28^-^OX40^+^ T cell subset in flow cytometry analysis. **a**, Gating strategy in PB samples of RA patients. **b**, Gating strategy in spleen samples of CIA mice. CD4^+^ T cells in a lymphocyte gate are divided into CD4^+^CD28^-^ and CD4^+^CD28^+^ T cells according to CD28 expression. OX40^+^ cells in a CD4^+^CD28^-^ T cell gate indicate the CD4^+^CD28^-^OX40^+^ T cell subset (red box). The IgG isotype controls and staining of OX40 are shown in the left and the right, respectively.


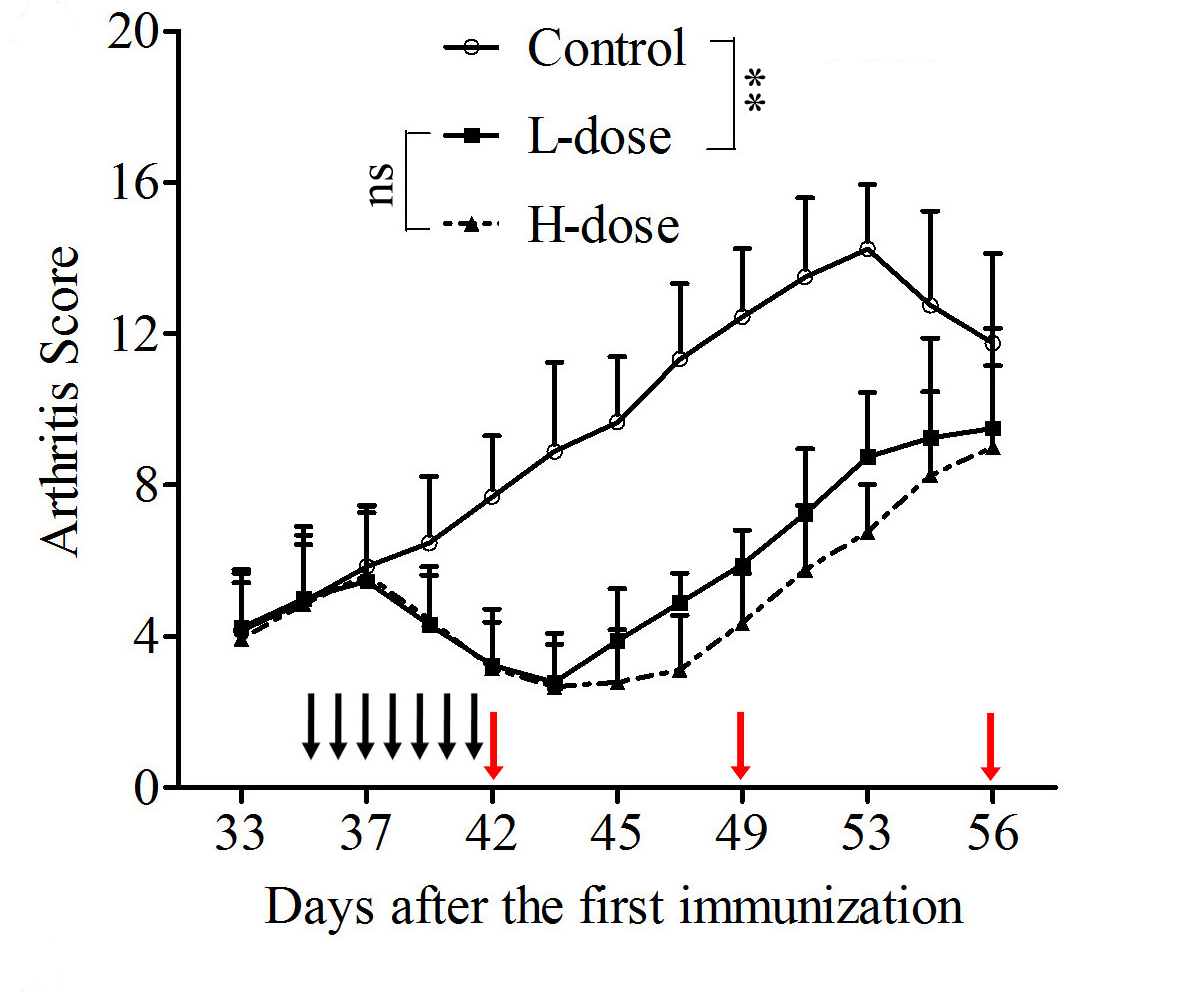


**Fig. S2.** Arthritic development in Dex-treated CIA mice. L-dose (n = 13) and H-dose (n = 13) Dex significantly suppressed the development of arthritis in CIA mice as compared with controls (n = 13). Black arrows indicate days of dosing, and red arrows indicate days when mice were sacrificed. ** *P* < 0.01, ns = not significant.

**Table S1. Clinical information about RA patients and control subjects**

| Group | RA | OA | HC |
| --- | --- | --- | --- |
| Sample size | 71 | 44 | 47 |
| Age | 53.51 ± 14.05 | 51.27 ± 16.51 | 50.02 ± 13.69 |
| Sex |  |  |  |
| Male | 18 | 11 | 11 |
| Female | 53 | 33 | 36 |
| Disease duration | 50.45 (1 – 240) | 52.48 (1 – 227) | - |
| Disease stage |  |  |  |
| Early RA (≤ 12 months) | 32 |  |  |
| Late RA (> 12 months) | 39 |  |  |
| Disease activity |  |  |  |
| Remission (DAS28 < 2.6) | 13 | - | - |
| Low (2.6 ≤ DAS28 ≤ 3.2) | 17 | - | - |
| Moderate (3.2 < DAS28 ≤ 5.1) | 27 | - | - |
| High (DAS28 > 5.1) | 14 | - | - |
| Disease manifestation |  |  |  |
| Extra-articular | 18 | - | - |
| Limited-joint | 53 | - | - |
| Drug use before study | - | - | - |

Sample size, sex, disease stage, activity and manifestation are the total number of subjects; age is presented in years ± (SD); disease duration is presented as the means (range of months); ‘–’ indicates ‘not applicable’.

**Table S2. Information about anti-human or anti-mouse antibodies and isotype controls**

|  | Clone | Conjugation | Supplier | Isotype |
| --- | --- | --- | --- | --- |
| Anti-human |  |  |  |  |
| CD4 | 13B8.2 | FITC | BECKMAN | IgG1, mouse |
| CD14 | RMO52 | FITC | BECKMAN | IgG2a, mouse |
| CD19 | J3-119 | FITC | BECKMAN | IgG1, mouse |
| OX40 | ACT35 | PE | BD Bioscience | IgG1, mouse |
| OX40L | 11C3.1 | PE | Biolegend | IgG1, mouse |
| CD28 | CD28.2 | PE-Cy5 | BD Bioscience | IgG1, mouse |
| Anti-mouse |  |  |  |  |
| CD4 | GK1.5 | FITC | eBioscience | IgG2b, rat |
| CD11b | M1/70 | FITC | eBioscience | IgG2b, rat |
| CD19 | eBio1D3 | FITC | eBioscience | IgG2b, rat |
| OX40 | OX86 | PE | Biolegend | IgG1, rat |
| OX40L | RM134L | PE | Biolegend | IgG2b, rat |
| CD28 | 37.51 | PE-Cy5 | Biolegend | IgG, syrian hamster |
| Isotype controls |  |  |  |  |
|  | MOPC-21 | FITC/PE/PE-Cy5 | BD Bioscience | IgG1, mouse |
|  | MOPC-173 | FITC | Biolegend | IgG2a, mouse |
|  | RTK4530 | FITC/PE | Biolegend | IgG2b, rat |
|  | RTK2071 | PE | Biolegend | IgG1, rat |
|  | SHG-1 | PE-Cy5 | Biolegend | IgG, Syrian Hamster |
